# RGMB enhances the suppressive activity of the monomeric secreted form of CTLA-4

**DOI:** 10.1038/s41598-019-43068-y

**Published:** 2019-05-06

**Authors:** Takashi Sekiya, Satoshi Takaki

**Affiliations:** 1 Section of Immune Response Modification, The Research Center for Hepatitis and Immunology, National Center for Global Health and Medicine, 1-7-1 Kohnodai, Ichikawa, Chiba 272-8516 Japan; 2 Department of Immune Regulation, The Research Center for Hepatitis and Immunology, National Center for Global Health and Medicine, 1-7-1 Kohnodai, Ichikawa, Chiba 272-8516 Japan

**Keywords:** Autoimmunity, Immune evasion

## Abstract

The immunoregulatory molecule CTLA-4 plays a crucial role in the maintenance of immune homeostasis. CTLA-4-neutralizing antibodies are now approved for the treatment of advanced melanoma, and are in development for treating other cancers as well. However, a thorough understanding of CTLA-4 function at the molecular level is necessary in order to develop strategies to prevent the unintended autoimmunity that is frequently associated with systemic blockade of CTLA-4 activity. Here, we describe an extracellular molecule, repulsive guidance molecule B (RGMB) as a novel binding partner of CTLA-4. RGMB expression was detected at high levels in dendritic cell subsets that have been suggested to have tolerogenic capabilities. RGMB binds an extracellular domain of CTLA-4, and specifically strengthens the binding of the monomeric, soluble form of CTLA-4 (sCTLA-4) to CD80, enhancing CTLA-4’s suppressive effect on co-stimulation. Examination of expression data from tumor tissues revealed a negative correlation between *RGMB* expression and immune activation status in the majority of non-hematologic tumor tissues. These findings advance our understanding of CTLA-4 activity, as well as identify the RGMB/CTLA-4 binding interface as a potential target for the development of novel immune checkpoint blockade therapies.

## Introduction

Optimal T cell priming requires not only stimulation through T cell antigen receptor (TCR), but also depends upon a second costimulatory signal mediated by the CD28 molecule on T cells binding CD80/86 on antigen presenting cells (APCs)^1^. CTLA-4 is a structural homolog of CD28 but acts as a negative regulator of co-stimulation by preventing CD28 from binding their shared ligands CD80/86, because it has a 20- to 40-fold higher affinity for CD80/86 than CD28 does^2–4^. The critical nature of CTLA-4’s influence on co-stimulation for the maintenance of immune homeostasis is demonstrated by the numerous autoimmune disorders that develop upon its abrogation in both mice and humans^5–8^. However, in tumor tissues, CTLA-4 often promotes unwanted repression of anti-tumor immunity in individuals suffering from cancer^9^.

CTLA-4 has emerged as one of the most successful targets in the field of the biologics^10,11^. For example, monoclonal antibodies specific for CTLA-4 that prevent CTLA-4 from binding CD80/CD86 dramatically activate anti-tumor immunity, leading to improved survival of patients with advanced melanoma^9^. In another example, CTLA-4-Ig, a fusion protein made up of the extracellular domain of CTLA-4 and the Fc portion of human IgG1, which binds to CD80/86 and disrupts co-stimulation, has been used to treat autoimmune diseases like rheumatoid arthritis^12^. However, those therapeutic agents generally do not possess specificity for the disease-causing co-stimulatory events, and thus significant adverse effects have been observed. In the case of CTLA-4 neutralizing antibodies, autoimmunity including colitis, dermatitis, and hepatitis, has occurred^13,14^, whereas CTLA-4-Ig treatment resulted in a higher incidence of respiratory infections^15,16^. Thus, a deeper understanding of CTLA-4’s function at the molecular level would guide the development of future, improved CTLA-4-targeted therapies.

The extracellular architecture of CTLA-4 is characterized by a single IgV-like domain that contains the CD80/86 ligand-binding site^17^. Of the several isoforms of CTLA-4, two isoforms include the extracellular ligand-binding domain^18^: in addition to the “full-length” isoform that contains a transmembrane domain, the other form of CTLA-4 is a secreted isoform that lacks the transmembrane domain^19^. The membrane-bound form of CTLA-4 (called “memCTLA-4” herein) contains a cysteine residue in the extracellular domain that mediates homo-dimerization and strengthens the affinity for CD80/86^20–22^. On the other hand, the secreted soluble form of CTLA-4 (called “sCTLA-4” herein) does not contain the cysteine residue and yields sCTLA-4 monomers^17,19^ that have a weaker affinity for CD80/86 than memCTLA-4. Thus, although serum concentrations of sCTLA-4 have been reported to be increased in various autoimmune disorders^23–27^, it is unclear whether the monomeric secreted form of sCTLA-4 is bioactive in patients.

The repulsive guidance molecule (RGM) family is comprised of the glycosyl-phosphatidylinositol (GPI)-anchored molecules RGMA, RGMB, and RGMC. Involvement of the RGM family in immune reactions has been suggested in several studies: first, anti-RGMA specific antibodies have been reported to attenuate clinical signs in experimental autoimmune encephalomyelitis^28^. The authors found that expression of RGMA was induced in BMDCs upon LPS stimulation, and treatment of CD4^+^ T cells with a recombinant RGMA protein enhanced Rap1 activity, which increases cells’ adhesion to ICAM-1. Second, RGMB-deficient mice die two to three weeks after birth^29^. Although the cause of mortality has not yet been clarified, a number of inflammatory cytokines, including IL-6, TNF-α, IL-1β, and IFN-γ were found to be upregulated in these mice. Third, it was shown that RGMB interacts with PD-L2 and mediates respiratory tolerance^30^.

In this report, we searched for a novel binding partner of CTLA-4 and identified that RGMB binds the extracellular domain of CTLA-4. Although RGMB did not enhance the repressive activity of memCTLA-4, it did specifically potentiate the activity of sCTLA-4. Furthermore, analysis of expression data sets from The Cancer Genome Atlas (TCGA) revealed a negative correlation between RGMB expression and various hallmarks of immune activation, in the majority of non-hematologic tumors. Our data suggest that the RGMB/CTLA-4 interaction interface is an intriguing target for novel immune checkpoint blockade therapies.

## Results

**Figure 1:**
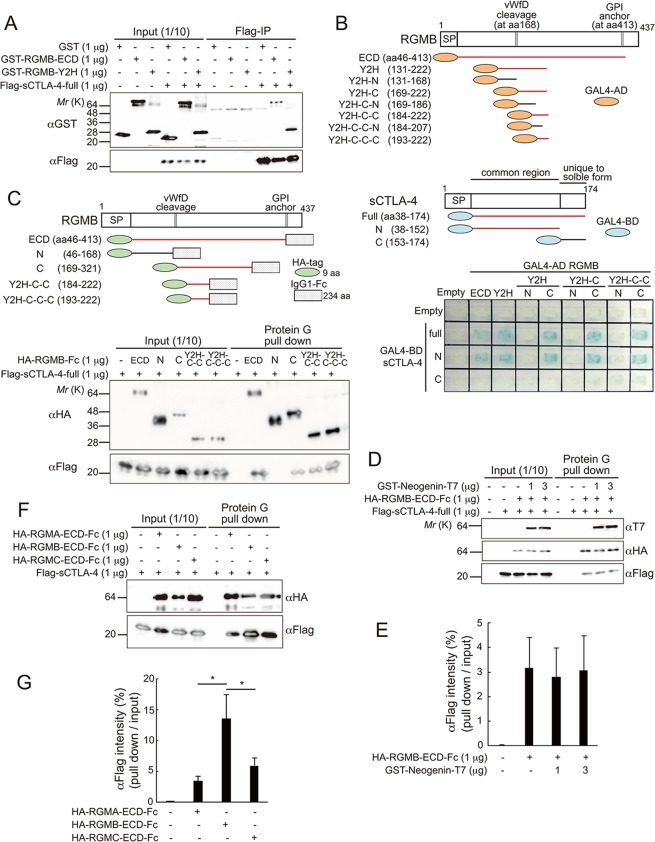
Identification of RGMB as a novel binding partner of CTLA-4. (**A**) Association of RGMB with sCTLA-4 *in vitro*. Mammalian cell-produced full-length sCTLA-4 (1 μg) was incubated with 1 μg of GST, GST-RGMB-ECD (extracellular domain), or GST-RGMB-Y2H (the residue that interacted with sCTLA-4 in yeast two-hybrid screening). Protein mixtures were immunoprecipitated with anti-Flag antibodies, then immunoblotted with the antibodies indicated. Full-length blots are shown in Supplementary Fig. S1A. (**B**) Mapping of RGMB and sCTLA-4 regions required for interaction. Deletion constructs of RGMB and sCTLA-4 were analyzed for their ability to interact with each other in yeast two-hybrid system. Regions that showed the interaction (indicated by the β-Galactosidase activity shown in the bottom panel) are red. (**C**) Association of mammalian cell-produced full-length sCTLA-4 and mammalian cell-produced Fc-fused RGMB fragments. Proteins were mixed in the indicated combinations, then pulled-down with protein G Sepharose. Bound proteins were immunoblotted with the antibodies indicated. Regions that showed interaction are red. Full-length blots are shown in Supplementary Fig. S1B. Blots for HA and Flag were cropped from the same gel of different parts, each with different exposure time. (**D**) Neogenin did not inhibit the interaction between RGMB and sCTLA-4 *in vitro*. Mammalian-cell-produced HA-RGMB-ECD-Fc and Flag-sCTLA-4 were incubated in the presence or absence of bacterial-produced Neogenin-T7. HA-RGMB-ECD-Fc was pulled-down with protein G Sepharose, and the bound proteins were immunoblotted with the antibodies indicated. Full-length blots are shown in Supplementary Fig. S2A. (**E**) Quantification of the results in (**D**). Intensities of the bands of pulled-downed Flag-sCTLA-4, relative to that of input bands, are shown. (**F**) Association of sCTLA-4 and each RGM family member *in vitro*. Mammalian cell-produced HA-RGM-ECD-Fc were incubated with mammalian cell-produced Flag-sCTLA-4, then pulled-down with protein G Sepharose. Bound proteins were immunoblotted with the antibodies indicated. Full-length blots are shown in Supplementary Fig. S2B. (**G**) Quantification of the results in (**F**). Intensities of the bands of pulled-downed Flag-sCTLA-4, relative to input bands, are shown. Scores show means ± SD of data obtained from three independent biological replicates. *p < 0.05 (one-way ANOVA with Bonferroni test).

We screened a universal human cDNA library with a yeast two-hybrid approach, using full-length human sCTLA-4 as bait. We isolated a fragment of the RGMB cDNA that encodes the middle part of the RGMB protein. Direct interaction between sCTLA-4 and RGMB was confirmed by co-precipitation of Flag-tagged recombinant human sCTLA-4 with a recombinant fusion protein of human RGMB fragments and glutathione-S-transferase (GST) (Fig. 1A). Two-hybrid assays with various deletion fragments of RGMB and sCTLA-4 identified a region (amino acids (aa) 193–222) that interacted with the extracellular domain of CTLA-4 (Fig. 1B). Interaction of this region with CTLA-4 was further confirmed with recombinant RGMB proteins produced in mammalian cells (Fig. 1C). This region of RGMB, aa193–222, comprises the fifth and sixth β strands and a linker region of the tightly packed β sandwich domain (aa158–296) of RGMB^31^. Since Neogenin, a well-known binding partner of RGMB, also interacts with this region^31,32^, we investigated whether Neogenin competes with CTLA-4 for binding to RGMB. As shown in Fig. 1D,E, the binding of CTLA-4 to RGMB was not disrupted by Neogenin, indicating that Neogenin and CTLA-4 bind different areas in the aa158–296 region of RGMB. Next, we analyzed the binding of other RGM family members, RGMA and RGMC, to CTLA-4. As shown in Fig. 1F,G, all RGM family members bound to CTLA-4; however, RGMB showed the strongest binding. Altogether, we identified RGMB as a novel binding partner of CTLA-4 and determined the binding regions that mediate this interaction.

**Figure 2:**
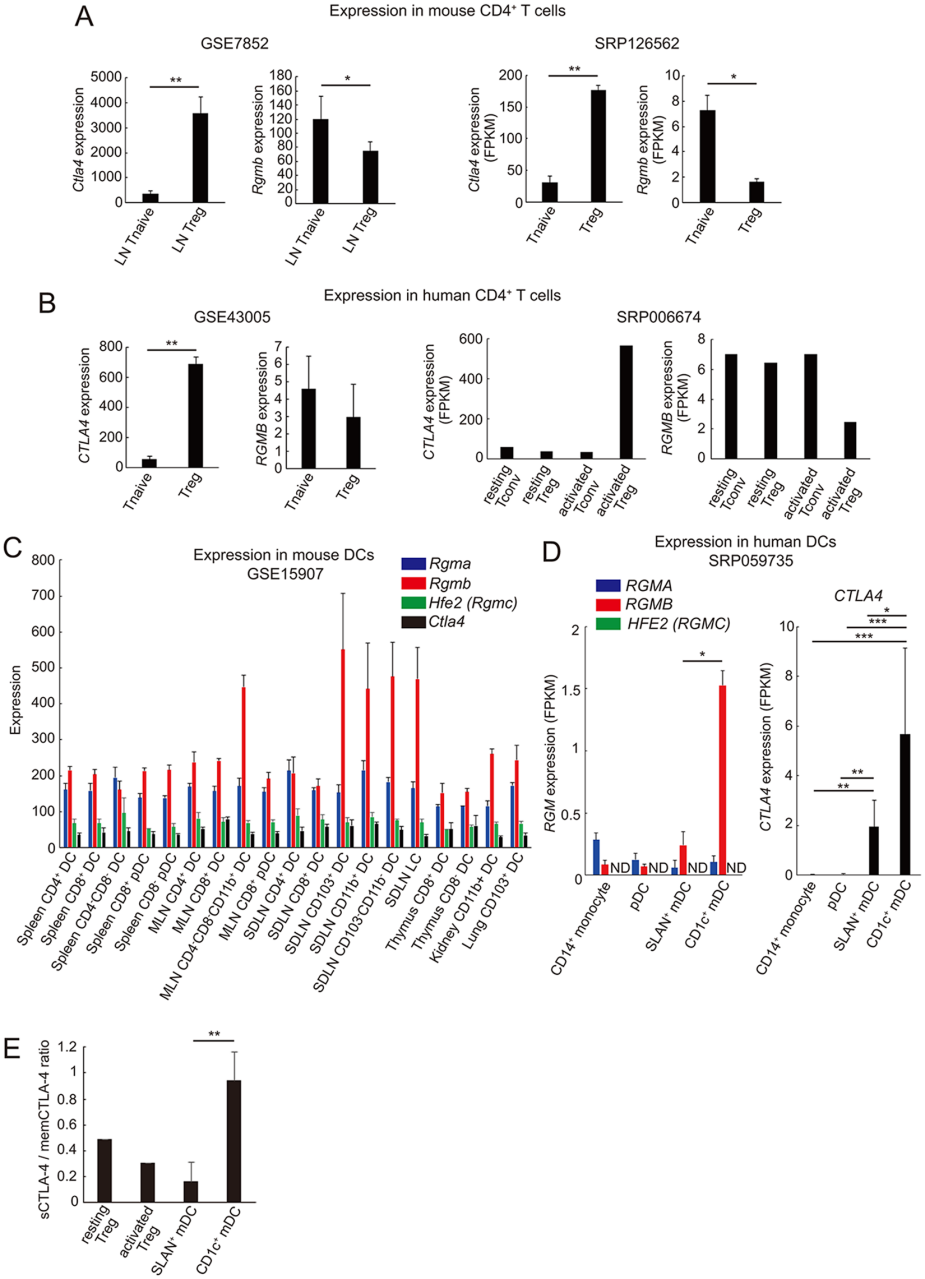
Expression profiles of *RGMB* and *CTLA4* in CD4^+^ T and dendritic cell subsets (**A**,**B**) Expression of *RGMB* and *CTLA4* mRNA in the indicated subsets of mouse (**A**) and human (**B**) CD4^+^ T cells. The microarray and the RNA-seq gene expression profiles with the indicated accession numbers were extracted from the Gene Expression Omnibus (GEO) database and the Sequence Read Archive (SRA) database, respectively. (**C**,**D**) Expression of *RGMB* and *CTLA4* mRNA in the indicated subsets of mouse (**C**) and human (**D**) dendritic cells. The microarray and the RNA-seq gene expression profiles with the indicated accession numbers were extracted from the Gene Expression Omnibus (GEO) database and the Sequence Read Archive (SRA) database, respectively. (**E**) Ratio of expression levels of soluble form of CTLA-4 (sCTLA-4) to that of membranous form of CTLA-4 (memCTLA-4) in the indicated cells. Expression levels of mRNA encoding each CTLA-4 variant in Treg cells and mDC were derived from RNA-seq data deposited in SRP006674 and SRP059735, respectively. *p < 0.05; **p < 0.01; ***p < 0.005 (Student’s t test (**A**,**B**,**E**), one-way ANOVA with Bonferroni test (**D**)).

Next, as CTLA-4 is primarily expressed by regulatory T (Treg) cells, and it exerts its suppressive activity by binding CD80/86 expressed on APCs, we analyzed the expression pattern of *RGMB* and *CTLA4* mRNA in various CD4^+^ T cell and dendritic cell (DC) subsets in both humans and mice. By examining publicly available datasets^33–38^, we found that although *CTLA4* expression was higher in Treg cells as expected, *RGMB* showed an opposite expression pattern to that of *CTLA4*, with lower expression in Treg cells in both humans and mice (Fig. 2A,B). Furthermore, the expression levels of *RGMB* were substantially lower throughout all of the CD4^+^ T cell subsets examined, compared with those of *CTLA4* (Fig. 2A,B). Thus, we concluded that it is less likely that RGMB and CTLA-4 act together on Treg cells. Next, we examined the expression of RGM family members and *CTLA4* in various dendritic cell subsets in both humans and mice. As shown in Fig. 2C, we found that *Rgmb* is expressed in various DC subsets in mice. Among them, CD11b^+^ DCs, CD103^+^ DCs, and Langerhans cells in tissue-draining lymph nodes, so called “tissue-migratory DCs”^39^, showed particularly high expression of *Rgmb*. Importantly, these “tissue-migratory DCs” have been shown to possess immune regulatory characteristics^35,40^. On the other hand, *Ctla4* expression was found to be low among all DC subsets examined in mice (Fig. 2C). In humans, as shown in Fig. 2D, expression of *RGMA* and *RGMB* was observed in all monocyte and DC subsets analyzed; however, *Hfe2* (which encodes RGMC) expression was not detected in any of the DC subsets. Particularly high expression of *RGMB* was observed in peripheral blood CD1c^+^ myeloid DC (mDC). Importantly, CD1c^+^ mDC have been suggested to possess tolerogenic characteristics^41,42^. Although CTLA-4 is primarily expressed by Treg cells, certain subsets of human DCs, including monocyte-derived mature DCs, have also been reported to express this molecule^43,44^. Accordingly, we detected *CTLA4* expression in both SLAN^+^ and CD1c^+^ mDC subsets (Fig. 2D). Importantly, compared with SLAN^+^ mDC cells, the CD1c^+^ mDC cells expressed higher amount of *CTLA4*. Furthermore, by independently analyzing the expression levels of the mRNA species that encode sCTLA-4 or memCTLA-4, it was found that CD1c^+^ mDC cells expressed the sCTLA-4 isoform at a significantly higher ratio, compared with SLAN^+^ mDC and Treg cells (Fig. 2E).

Collectively, based the expression pattern of *RGMB* and *CTLA4*, we concluded that RGMB does not work on Treg cells, but is however expressed in certain subsets of DCs with regulatory characteristics. On these DCs, RGMB interacts with CTLA-4 expressed by Treg cells or by the same RGMB-expressing DCs in an autocrine manner. In addition, as *RGMB* expression was consistently higher among the RGM family members in DCs, and also RGMB showed the strongest binding affinity to CTLA-4 in Fig. 1F,G, we decided to focus on this molecule in subsequent experiments.

**Figure 3:**
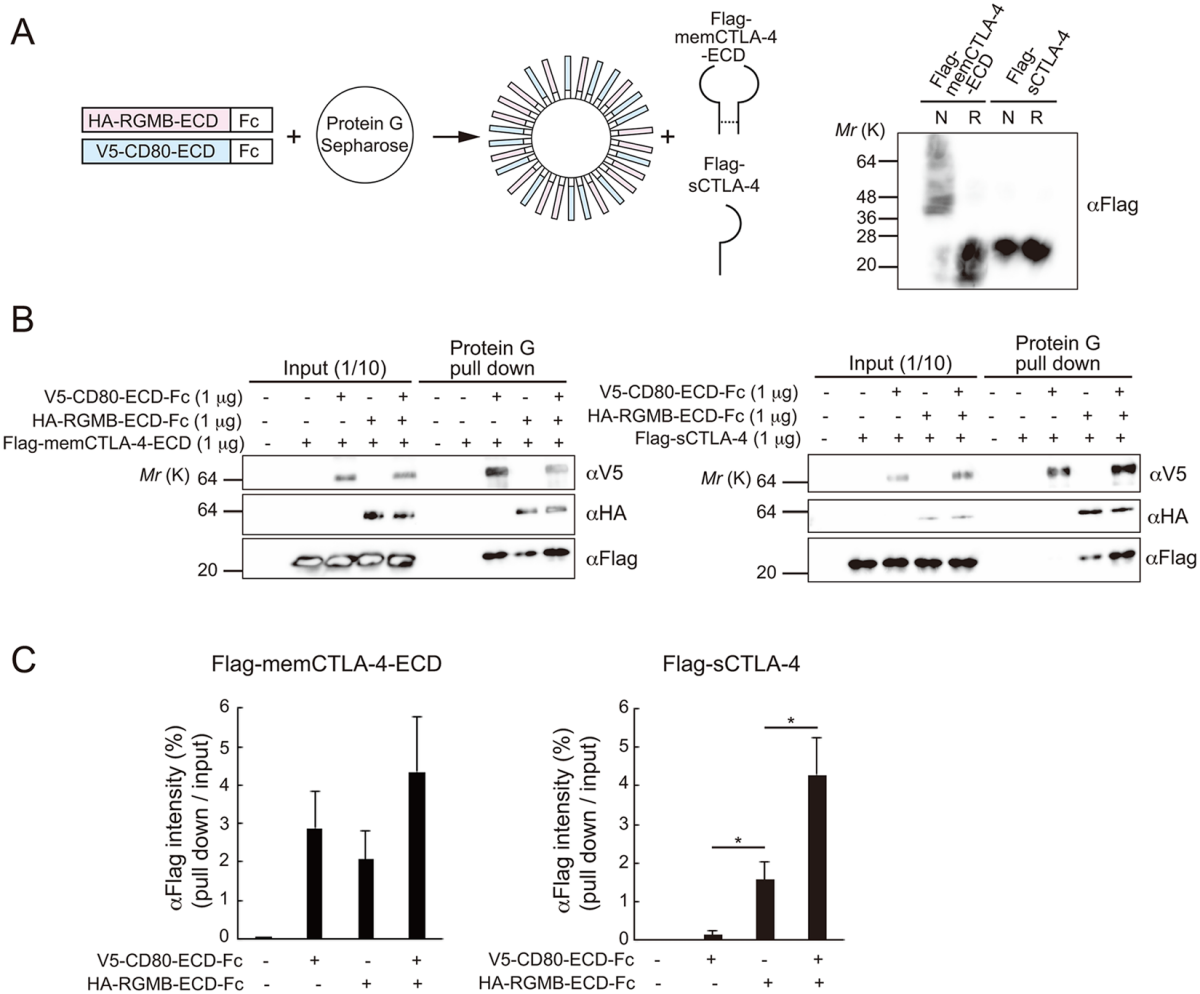
RGMB strengthens the binding of sCTLA-4 to CD80. (**A**) Left: A schematic of experiments shown in (**B**). Right: Western blot analysis of 10 ng of mammalian cell-produced Flag-tagged extracellular domain of the membrane-bound CTLA-4 (Flag-memCTLA-4-ECD) or Flag-tagged sCTLA-4 under non-reducing (N) and reducing (R) conditions. Full-length blots are shown in Supplementary Fig. S3A. (**B**) Binding of Flag-memCTLA-4-ECD (left) or Flag-sCTLA-4 (right) to protein G Sepharose loaded with or without HA-RGMB-ECD-Fc and/or V5-CD80-ECD-Fc. Proteins pulled-downed with protein G Sepharose were analyzed by 15% SDS-PAGE and immunoblotted with the antibodies indicated. Full-length blots are shown in Supplementary Fig. S3B. Blots for V5 and Flag were cropped from different parts of the same gel, and HA blots were cropped from different gel, each with different exposure time. (**C**) Quantification of the results in (**B**). Intensities of the bands of pulled-down Flag-CTLA-4, relative to the intensities of the input bands, are shown. Scores show means ± SD of data obtained from three independent biological replicates. *p < 0.05 (one-way ANOVA with Bonferroni test).

Next, we investigated the functional output of the interaction between RGMB and CTLA-4. From the expression analysis above, it was suggested that RGMB is expressed on antigen presenting cells (APCs) where it mediates the inhibitory activities of CTLA-4. Thus, we set up an *in vitro* biochemical experiment, shown in Fig. 3A, to assess the effects of RGMB on the interaction between CTLA-4 and CD80. In this experiment, we first mixed Fc-fused extracellular domains of RGMB and/or CD80 with protein G Sepharose, thus making particles that mimic cells that present RGMB and/or CD80 on their surface. Next, those particles were incubated with recombinant sCTLA-4 or the extracellular domain of memCTLA-4 (memCTLA-4-ECD), after which the binding of CTLA-4 to the particles was analyzed. We confirmed that memCTLA-4-ECD and sCTLA-4 exist in dimeric and monomeric forms, respectively (Fig. 3A). As shown in Fig. 3B,C, in the absence of RGMB, memCTLA-4-ECD showed strong binding affinity for CD80, whereas sCTLA-4 exhibited poor binding affinity for CD80, as expected. In contrast, in the presence of both RGMB and CD80, sCTLA-4 exhibited equivalent binding affinity for CD80 to that of memCTLA-4-ECD (Fig. 3B,C). Collectively, the above biochemical experiments and the expression profiling data strongly suggest that RGMB expressed on APCs strengthens the intrinsically weak interaction between sCTLA-4 and CD80.

**Figure 4:**
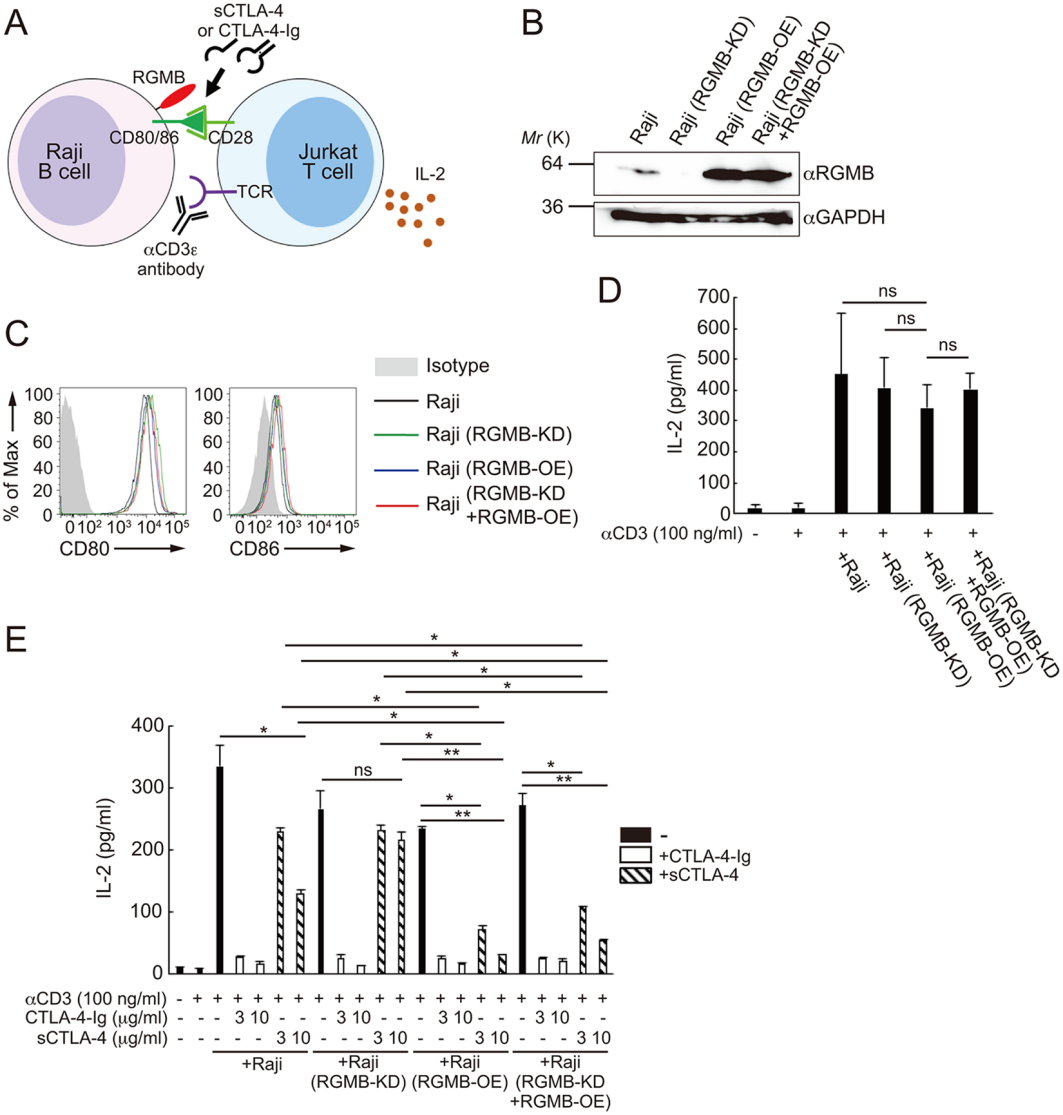
RGMB strengthens the suppressive ability of sCTLA-4 on co-stimulation. (**A**) A schematic of experiments shown in (**E).** **(B**) Western blot analysis of the expression levels of RGMB and GAPDH. Cell lysates were prepared from unmodified Raji cells, Raji cells in which RGMB expression was knocked-down by co-expression of dCas9-KRAB and the sgRNA targeting the *RGMB* promoter (Raji (RGMB-KD)), Raji cells in which RGMB was retrovirally overexpressed (Raji (RGMB-OE)), or Raji cells in which knocked-down RGMB expression was rescued by retroviral overexpression of RGMB (Raji (RGMB-KD + RGMB-OE)). Full-length blots are shown in Supplementary Fig. S4. Blots for GAPDH and RGMB were cropped from different parts of the same gel, with different exposure times. (**C**) Flow cytometry for the indicated molecules in Raji, Raji (RGMB-KD), Raji (RGMB-OE), Raji (RGMB-KD + RGMB-OE) cells (black, green, blue, and red lines, respectively). Shaded area represent the isotype-matched control. (**D**) ELISA analysis of IL-2 concentrations in culture supernatants. Jurkat T cells were left unstimulated or stimulated for 24 hr by incubation with anti-CD3ε antibodies (OKT-3, 100 ng/ml) and Raji B cells, which were unmanipulated or manipulated as in (**B).** **(E**) ELISA analysis of IL-2 concentrations in culture supernatants. Jurkat T cells were left unstimulated or stimulated for 24 hr by incubation with anti-CD3ε antibodies (OKT-3, 100 ng/ml) and Raji B cells, which were unmanipulated or manipulated as in (**B**). Raji cells were pre-incubated with or without CTLA-4-Fc (3 or 10 μg/ml) or sCTLA-4 (3 or 10 μg/ml) for 30 min. Results shown in (**D**,**E**) are representative of three **i**ndependent biological replicates, each performed in triplicate, means ± SD. *p < 0.05; **p < 0.01; ns, not significant (one-way ANOVA with Bonferroni test).

Next, we investigated the effects of RGMB on CTLA-4’s ability to suppress co-stimulation. In this experiment, we employed a well-known co-culture system using Raji B lymphoma cells and Jurkat T lymphoma cells (Fig. 4A). In this experimental system, combining TCR stimulation by anti-CD3ε antibodies and co-stimulation by CD80 present on Raji cells activated the Jurkat cells. We monitored the activation status of the Jurkat cells by IL-2 expression. First, we generated Raji cells that differentially expressed RGMB (Fig. 4B). In order to repress endogenous RGMB expression in Raji cells, we employed the CRISPR interference (CRISPRi) system, in which a fusion protein consisting of the transcriptional repressive domain of ZNF10 (KRAB) and nuclease-deficient Cas9 (dCas9) is recruited to a specific genomic region by single guide RNA (sgRNA), resulting in the repression of a specific gene of interest^45^. As shown in Fig. 4B, co-expression of dCas9-KRAB with an sgRNA targeting the *RGMB* promoter suppressed endogenous RGMB expression in Raji cells (denoted as “RGMB-KD”). In order to overexpress RGMB, we retrovirally transduced *RGMB* cDNA into Raji cells (denoted as “RGMB-OE”). In addition, we constructed Raji cells in which CRISPRi-mediated RGMB knockdown of endogenous RGMB was rescued by retroviral transduction of RGMB (denoted as “RGMB-KD + RGMB-OE”). First, we confirmed that differential expression of RGMB did not affect CD80 and CD86 expression levels *per se* in Raji cells (Fig. 4C).

We observed that the Jurkat cells were activated when they were stimulated with anti-CD3ε antibodies and co-stimulation was provided by the Raji cells (Fig. 4D). In addition, as expected from the equivalent CD80 and CD86 expression levels, differential expression levels of RGMB did not affect the co-stimulatory ability of Raji cells *per se* (Fig. 4D). CTLA-4-Ig, an IgG Fc fusion homodimer of the extracellular domain of CTLA-4, which is currently approved therapeutic agent for rheumatoid arthritis, substantially inhibited IL-2 secretion from Jurkat cells, regardless of RGMB expression levels (Fig. 4E). This observation confirms that the dimeric, potentiated form of CTLA-4 does not require RGMB to exert its repressive activity. Conversely, monomeric sCTLA-4 exhibited weak suppressive activity on un-modified Raji cells. However, on Raji cells that overexpressed RGMB, sCTLA-4 exhibited a substantial suppressive ability over Jurkat activation (Fig. 4E). Furthermore, the residual suppressive activity of sCTLA-4 on unmodified Raji cells was abrogated by CRISPRi-mediated knockdown of RGMB (Fig. 4E). The effects obtained with the CRISPRi-mediated manipulation was not an off-target effect, as RGMB expression rescued the suppressive ability of sCTLA-4 on Jurkat co-activation (Fig. 4E). Collectively, our findings indicate that RGMB on APCs strengthens the suppressive ability of sCTLA-4, which itself does not possess significant intrinsic suppressive activity.

**Figure 5:**
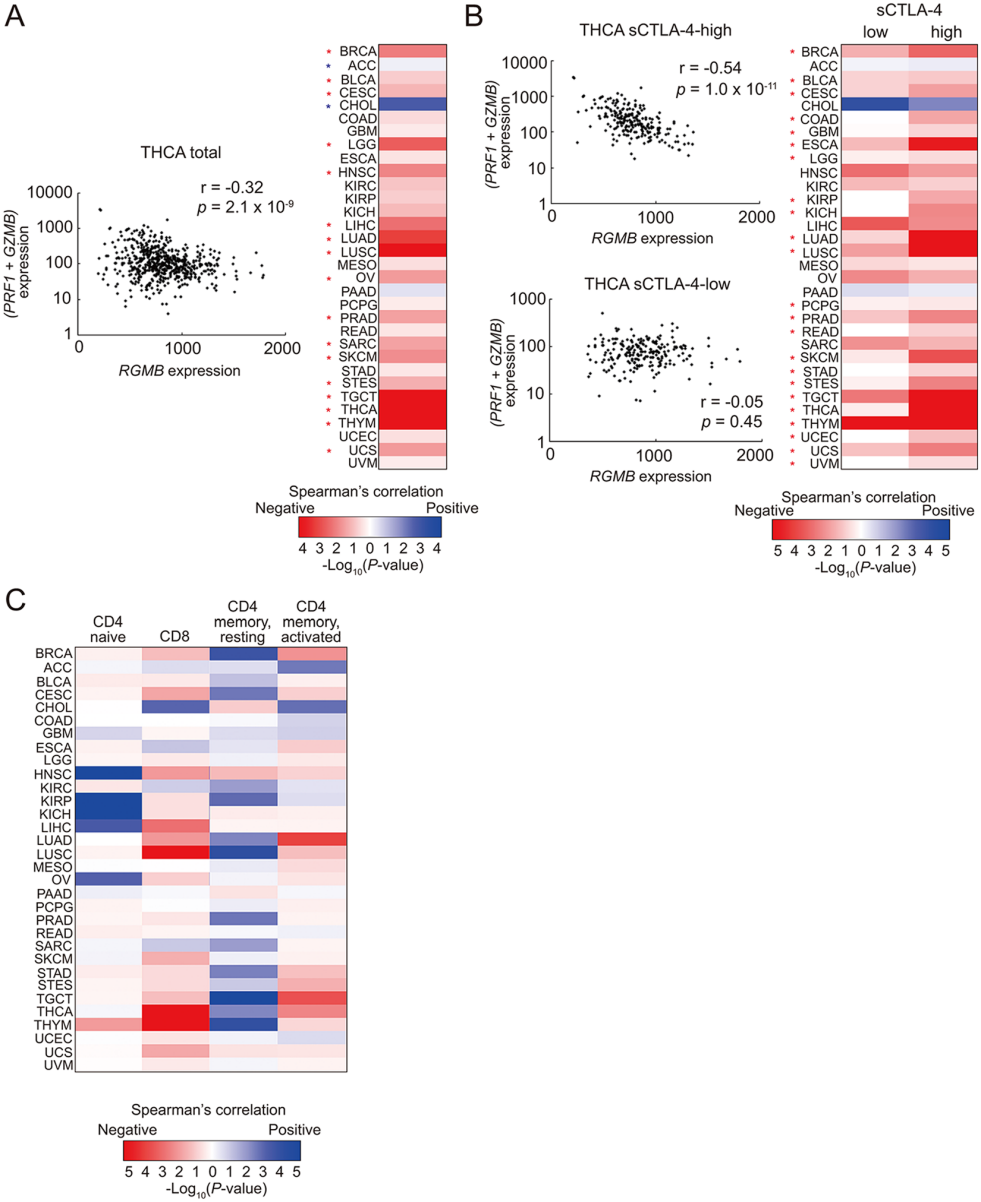
RGMB expression is negatively correlated with the hallmarks of immune activation. (**A**) Correlation of *RGMB* and *GZMB* plus *PRF1* mRNA levels in the indicated cancers. The result obtained with the thyroid carcinoma (THCA) dataset is shown on the left as an example. Red and blue asterisks indicate significant (p < 0.05) negative and positive correlations, respectively. For a complete list of the TCGA cancer type abbreviations, please refer to https://gdc.cancer.gov/resources-tcga-users/tcga-code-tables/tcga-study-abbreviations. (**B**) Effects of sCTLA-4 expression levels on the correlation between *RGMB* and *GZMB* plus *PRF1* mRNA levels observed in (**A**). Samples in each cancer type were divided into halves, according to the expression levels of sCTLA-4 that were determined by the method shown in Supplementary Fig. S6. Cancer types that showed a greater significant negative correlation between *RGMB* levels and *GZMB* plus *PRF1* levels in the sCTLA-4-high subset than in the sCTLA-4-low subset are labeled by red asterisks. The result obtained with the thyroid carcinoma (THCA) dataset is shown on the left as an example. (**C**) Correlation between the percentages of the indicated T cell populations, which were determined by CIBERSORT, and *RGMB* mRNA expression in the indicated cancer types.

Elevated expression levels of CTLA-4 are often observed in advanced tumor tissues, and blockade of CTLA-4 function frequently reactivates anti-tumor immunity. Therefore, we next investigated the roles of RGMB in anti-tumor immunity. We examined RNA-seq data of thirty-three non-hematological cancer types from TCGA database. By estimating the cytotoxic T lymphocyte (CTL) activities through Perforin (encoded by *PRF1*) and Granzyme B (encoded by *GZMB*) expression, we observed a negative correlation between *RGMB* expression levels and CTL activities in 30 of 33 cancer types (*p* = 2.6 × 10^−6^, χ2 test, Fig. 5A). Among the 30 cancer types that exhibited that negative correlations, 17 reached statistical significance (*p* < 0.05).

Given that we demonstrated that RGMB specifically supports the suppressive activity of sCTLA-4, we next investigated to what extent the amount of sCTLA-4 affected the negative correlations observed between *RGMB* expression and CTL activity in various cancer tissues. In this analysis, we determined the expression levels of sCTLA-4 as: expression levels of total *CTLA4* multiplied by the proportion of sCTLA-4. As the membrane-bound and soluble forms of CTLA-4 are determined by alternative splicing (i.e., the membrane-bound form includes exon 3, but the soluble form does not, Supplementary Fig. S6A), we derived the proportion of sCTLA-4 from the reads count of exons 2, 3, and 4. Proportions of splicing variants are usually determined from the read counts at the exon-exon junctions (“Method 1” in Supplementary Fig. S6B). However, the majority of samples in TCGA data sets have “0” exon:exon junction read counts, and thus are unsuited for our purpose. Therefore, we developed a way to calculate the proportion of sCTLA-4 from the reads that were mapped to the exon bodies (“Method 2” in Supplementary Fig. S6B). As shown in Supplementary Fig. S6C, since we observed a significant positive correlation between the proportion of sCTLA4 estimated by “Method 1” and that estimated by “Method 2”, we decided to employ “Method 2” for deriving the proportion of sCTLA-4 for our subsequent analyses. After separating the samples into sCTLA-4-high and -low groups for each cancer type, correlations between *RGMB* expression and CTL activity were found to be more significant in the sCTLA-4-high groups than in the sCTLA-4-low groups in 23 cancer types, out of the 30 cancer types that showed a negative correlation between *RGMB* expression and CTL activity above ((*p* = 0.004, χ^2^ test, Fig. 5B).

Next, we conducted a lymphocyte subset analysis of the tumor-infiltrating lymphocytes (TIL) to analyze the correlation between RGMB expression levels and particular lymphocyte subsets in tumor tissues. In this analysis, we inputted TCGA data sets into the CIBERSORT algorithm, which can estimate the abundance of different immune cell types in mixed cell populations from gene expression data^46^. As shown in Fig. 5C, *RGMB* expression levels negatively correlated with CD8 T cell abundance (25 of 33 cancers, *p* = 0.003), as well as with activated memory CD4^+^ T cells (26 of 33 cancers, *p* = 0.001). Conversely, *RGMB* levels positively correlated with resting memory CD4^+^ T cells (28 of 33 cancers, *p* = 2.6 × 10^−4^).

Altogether, our findings show that expression levels of RGMB negatively correlate with multiple hallmarks of immune activation, such as CTL effector gene expression and abundance of activated T cells, in tumor tissues. In addition, our data suggest that the suppressive effect of RGMB on anti-tumor immunity is mediated by sCTLA-4.

**Figure 6:**
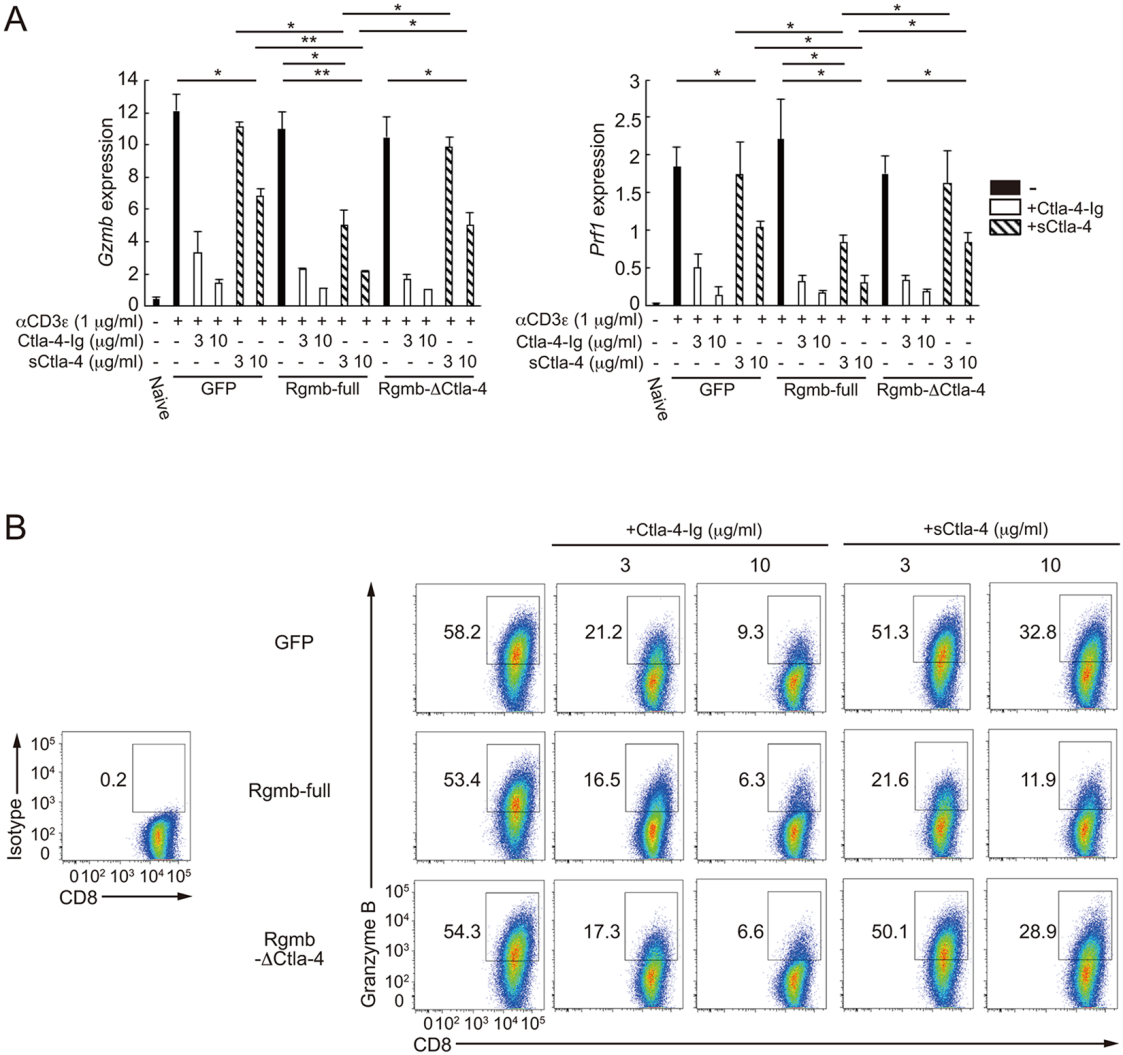
RGMB strengthens the suppressive ability of sCTLA-4 on co-stimulation of CD8^+^ T cells (**A**) qRT-PCR analysis of *Gzmb* and *Prf1* expression in Naive CD8^+^ T cells or in mixed lymphoid culture of CD8^+^ T cells and BMDCs constructed as in Supplementary Fig. S7. BMDCs were pre-incubated with or without Ctla-4-Fc (3 or 10 μg/ml) or sCtla-4 (3 or 10 μg/ml) for 30 min. Result shown are presented relative to *Hprt* expression. (**B**) Flow cytometry of Granzyme B and CD8 expression in mixed lymphoid culture of CD8^+^ T cells and BMDCs constructed as in Supplementary Fig. S7. The left panel shows a control staining with isotype IgG for Granzyme B staining shown on the right. In the mixed lymphoid culture in (**A**,**B**), naive CD8^+^ T cells were left stimulated for 6 days by incubation with anti-CD3ε antibodies (145-2C11, 1 μg/ml) and BMDCs. BMDCs were pre-incubated with or without Ctla-4-Fc (3 or 10 μg/ml) or sCtla-4 (3 or 10 μg/ml) for 30 min. Results shown in (**A**) are representative of two independent biological replicates, each performed in triplicate, means + SD. *p < 0.05; **p < 0.01; (one-way ANOVA with Bonferroni test).

Our results suggest a model that RGMB strengthens the suppressive activity of sCTLA-4 on co-stimulation, repressing CD8^+^ T cell-mediated killing activities in tumor tissues. However, as NK cells are also capable of exerting tumor cell killing activity by Granzyme B and Perforin expression, we next determined whether RGMB actually augments the repressive activity of sCTLA-4 on co-stimulation of CD8^+^ T cells. In this experiment, we analyzed the effect of RGMB expression levels in DCs on the suppressive ability of sCTLA-4 on co-stimulation of CD8^+^ T cells by mixed lymphoid reaction (MLR) (Supplementary Fig. S7A). As mouse cells and molecules were used in this experiment, we first confirmed the interaction between mouse Rgmb and mouse Ctla-4 (Supplementary Fig. S7B,C). This interaction was substantially attenuated by deletion of the region (aa196–225) that corresponds to the CTLA-4 binding site in human RGMB (denoted as “Rgmb-ΔCTLA-4”: residual interaction with Ctla-4 could possibly be caused by the existence of other weak binding sites or by a detection of a newly generated structure in the deletion mutant) (Supplementary Fig. S7B,C). Then, we generated bone marrow derived dendritic cells (BMDCs) that overexpress either full-length Rgmb or Rgmb-ΔCTLA-4 by retroviral transduction (Supplementary Fig. S7A,D,E). We found that overexpression of either protein did not affect the expression levels of CD80 and CD86 in BMDC *per se* (Supplementary Fig. S7F). By performing MLR using BMDCs generated as described above and naive CD8^+^ T cells, we found that *Gzmb* and *Prf1* expression was substantially induced in the simultaneous presence of TCR stimulation by anti-CD3ε antibody and co-stimulation by BMDCs (Fig. 6A). CTLA-4-Ig equally efficiently suppressed *Gzmb* and *Prf1* expression, regardless of the expression levels of Rgmb in BMDCs. On the other hand, the suppressive activity of sCTLA-4 on *Gzmb* and *Prf1* expression was significantly augmented on BMDCs that overexpressed full-length Rgmb, compared with those on control BMDCs (Fig. 6A). These observations were also recapitulated at the protein level for Granzyme B expression (Fig. 6B). Collectively, it was confirmed that RGMB augments the repressive ability of sCTLA-4 on co-stimulation of CD8^+^ T cells.

## Discussion

The soluble form of CTLA-4 has been reported to be upregulated in various disease settings. However, the significance of these findings has been unclear, as this form of CTLA-4 does not show strong affinity for CD80/86, and thus is presumed to exert a weak repressive effect on co-stimulation. In this study, we identified the extracellular molecule RGMB as a novel binding partner of CTLA-4. Our data suggest that RGMB is expressed on APCs and interacts with the extracellular domain of CTLA-4. This interaction specifically strengthens the binding of sCTLA-4 with CD80, enhancing its suppressive effect on co-stimulation. We further found that expression levels of RGMB negatively correlated with various hallmarks of immune activation in tumor tissues, including the expression of CTL effector molecules.

Involvement of RGMB in immune suppression has been suggested by the upregulation of various inflammatory cytokines in RGMB-deficient mice^29^. So far, several mechanisms have been reported to mediate RGMB’s effects on immune repression. First, RGMB expression on RAW264.7 cells was shown to augment bone morphogenetic protein (BMP)-mediated suppression of IL-6 expression^29^. Second, it was shown that RGMB interacts with PD-L2 and mediates respiratory tolerance^30^. Currently, we do not know whether each mechanism independently regulates specific immunological events or if there is cooperation among them to regulate common events. However, augmentation of sCTLA-4 activity is expected to be a major mechanism for RGMB to suppress anti-tumor immunity, as it was shown that the negative correlation between *RGMB* expression and CTL activity in tumor tissues was more evident with higher levels of sCTLA-4.

RGMB enhanced the suppressive activity specifically of sCTLA-4, and not that of memCTLA-4. This reflects the fact that monomeric sCTLA-4 poorly binds CD80, whereas dimeric memCTLA-4 possesses a much greater binding affinity for CD80. The stable complex formed between dimeric CTLA-4 and CD80 was explained by the crystal structure of the CD80/CTLA-4 complex: CTLA-4 and CD80 homodimers pack together to form a periodic arrangement in which bivalent CTLA-4 homodimers bridge bivalent CD80 homodimers, thus generating a stable “skewed zipper” arrangement^21,22^. Two mechanisms potentially explain our observation that RGMB strengthened the interaction between sCTLA-4 and CD80 in our biochemical experiments: first, RGMB simply increased local concentrations of sCTLA-4 near the surface of the CD80-coated beads. Second, and not mutually exclusive, as the Fc-fusion proteins primarily form homodimers, the homodimeric Fc-fused RGMB “bundled” sCTLA-4 molecules, which potentially mimics the effects of CTLA-4 dimerization. Regarding this possibility, it has been reported that RGMC primarily exists as a monomer^32^. Thus, the native form of RGMB may not posses the ability to “bundle” sCTLA-4. However, it has been reported that BMPs, well-known RGMB’s binding partners, primarily exist as dimers^47^, and also Neogenin, another RGMB binding partner, forms oligomers under certain circumstances^31^. Thus, these oligomeric binding partners are supposed to accommodate multiple RGMB molecules as higher order complexes, thus closely co-locating RGMB/sCTLA-4 units. In this regard, we observed that an optimal level of sCTLA-4’s suppression on Raji cell-mediated co-stimulation of Jurkat cells was achieved only after pre-incubation (>30 min) of Raji cells with sCTLA-4 (data not shown). This suggests that the higher order complexes between RGMB/CTLA-4 units and RGMB’s binding partners are formed during the pre-incubation process. Whether BMPs, Neogenin, or an unidentified interaction partner mediate the formation of such higher order structures should be clarified in future studies. However, this model is likely, as Neogenin did not compete with sCTLA-4 for RGMB binding (Fig. 1D,E), and also BMPs bind a domain on RGMB that is distant from the CTLA-4 binding site^47^.

RGMB expression was observed in various DC subsets, and its expression tended to be higher in CD103^+^, CD11b^+^ DCs, and Langerhans cells in tissue-draining lymph nodes in mice. These DC subsets are understood to be “tissue-migratory classical DCs (cDCs)” that have migrated from the drained tissues, distinguished from “lymph node-resident DCs” that are composed of pDCs, CD8^+^ cDCs and CD8^-^ cDCs^39^. Importantly, compared with the lymph node-resident DCs, tissue-migratory cDCs have been shown to express immunomodulatory genes that potentially dampen T cell activation^35,40^. Furthermore, tissue-migratory cDCs were reported to positively regulate Treg cell responses in the steady state^40^. In human cells, while RGMB expression was detected in all mDC subsets examined, its expression was substantially higher in the CD1c^+^ mDC subset, compared with the SLAN^+^ mDC subset. Although the functional classification of human DCs according to surface marker expression has been a subject of much debate, several reports have implicated tolerogenic and inflammatory characteristics in CD1c^+^ and SLAN^+^ mDCs, respectively^41,42^. Collectively, the expression profiling of various DC subsets revealed higher RGMB expression in the potentially tolerogenic DC subsets, in both humans and mice. In addition, we also found that CD1c^+^ mDC themselves express substantial amounts of CTLA-4, particularly in its soluble form. CTLA-4 expression has been detected in human DCs, including monocyte-derived mature cell subset^43,44^. CTLA-4 on human DCs has been reported to transmit signals that induce IL-10 and repress IL-8/IL-12^43^, and to inhibit maturation of DCs^44^. In addition to these signaling-mediated suppressive events by memCTLA-4, we propose a model that sCTLA-4 acts in autocrine manner, with the help of RGMB expressed on the same cell, to mediate the suppressive ability of tolerogenic DCs.

It is commonly accepted that the membrane-bound form, but not the soluble form, primarily mediates the immunoregulatory roles of CTLA-4 under homeostatic conditions. On the other hand, as selective reduction of the soluble form of CTLA-4 is associated with tissue-specific autoimmunity both in mice and humans, it is speculated that sCTLA-4 has a tissue- and/or a circumstance-specific regulatory role^8,48^. In addition, elevated levels of sCTLA-4 were detected in the sera of patients with various autoimmune diseases^23–26^. The significance of these phenomena has not been clarified. However, future studies involving both sCTLA-4 and RGMB will reveal the significance of sCTLA-4 in such disease settings. In the majority of tumors, we observed a negative correlation between RGMB expression levels and the hallmarks of immune activation, and this correlation was further strengthened by sCTLA-4 expression. Our data suggest that the interaction surface between RGMB and sCTLA-4 presents a novel therapeutic target for both autoimmune diseases and tumors, especially in cases where systemic alteration of CTLA-4 induces adverse effects.

## Methods

### Antibodies

Anti-Flag M2 monoclonal (SIGMA), anti-HA monoclonal (12CA5, Roche), anti-V5 monoclonal (OZA3, MBL), anti-T7 polyclonal (PM022, MBL), anti-GST monoclonal (GT5, MBL), anti-human CD3ε monoclonal (OKT3, TONBO Biosciences), anti-mouse CD3ε monoclonal (145-2C11, TONBO Biosciences), anti-RGMB polyclonal (ab96727), Alexa Fluor 647-conjugated anti-human CD86 monoclonal (IT2.2, BioLegend), PE/Cy7-conjugated anti-human CD80 monoclonal (2D10, BioLegend), APC-conjugated anti-mouse CD80 monoclonal (16-10A1, TONBO Biosciences), PE-conjugated anti-mouse CD86 monoclonal (GL-1, TONBO Biosciences), APC-Cy7-conjugated anti-mouse CD8 monoclonal (53-6.7, TONBO Biosciences), PE-Cy7 conjugated anti-mouse CD11c monoclonal (N418, TONBO Biosciences), and Alexa Fluor 647-conjugated anti-mouse Granzyme B (GB11, BioLegend) antibodies were commercially obtained.

### Yeast two-hybrid screening

For construction of bait, the ORF of full-length human sCTLA-4, that does not contain the signal peptide coding region, was PCR-amplified and sub-cloned into pGBK-T7 plasmid (Takara Clontech). Using the bait plasmid, sCTLA-4 binding proteins were screened against Mate & Plate^TM^ Library - Universal Human (Normalized) (Takara Clontech), according to the manufacture’s protocol.

### Yeast two-hybrid analysis for domain mapping

For the mapping of regions responsible for the RGMB and CTLA-4 interaction by yeast two-hybrid method, human RGMB cDNA and human sCTLA-4 cDNA were sub-cloned into pGADT7 and pGBK-T7 plasmids respectively. Then, each combination of plasmids were transformed into Y2H-Gold yeast strain (Takara Clontech) by lithium acetate method and were spread onto Synthetic Defined (SD) agar plate that did not contain tryptophan and leucine (SD -TL). Then, single colonies was picked up from the each plate, and streaked onto SD -TL agar plate supplemented with X-αGal (20 μg/ml).

### Protein purification from bacteria

For purification of bacterial-produced GST-fusion proteins, including GST, GST-RGMB-ECD, GST-RGMB-Y2H, and GST-Neogenin-T7, corresponding pGEX (GE Healthcare)-based plasmids were transformed into Rosseta *E*. *coli* strain (Novagen). 500 ml of the bacteria culture were grown until OD600 reached 0.6, then protein production were induced by an addition of IPTG to 0.5 mM. After 3 hour incubation at 18 °C with shaking, bacteria were harvested and sonicated, thus making crude extracts of the recombinant proteins. Then, the crude protein extracts were purified with Glutathione Sepharose (GE Healthcare).

### Protein purification from mammalian cells

For purification of His_6_-tagged proteins, including Flag-sCTLA-4-full, Flag-memCTLA-4-ECD, and mouse Flag-sCtla-4-full, CMV promoter-driven expression plasmids were transfected into 293FT cells. Twelve hours after transfection, cells were collected by trypsinization and moved to a spinner flask that contain 200 ml of serum free media BalanCD HEK293 (IrvineScientific), then cultured at 37 °C, 5%CO_2_ under constant agitation with magnetic stirrer for three days. Culture supernatants were collected and dialyzed twice against 1 L of PBS. Recombinant proteins were purified from the dialyzed supernatant with 1 ml of Ni-NTA agarose (Qiagen), according to the manufacture’s protocol. After elution from Ni-NTA agarose with 500 μl of imidazole, protein solutions were dialyzed twice against 100 ml of PBS. For purification of Fc-fusion proteins, including HA-RGMB-ECD-Fc, HA-RGMB-Nterm-Fc, HA-RGMB-Cterm-Fc, HA-RGMB-Y2H-C-C-Fc, HA-RGMB-Y2H-C-C-C-Fc, HA-RGMA-ECD-Fc, HA-RGMC-ECD-Fc, V5-CD80-ECD-Fc, CTLA-4-Ig, and mouse Ctla-4-Ig, CMV promoter-driven expression plasmids were transfected into 293FT cells. Twelve hours after transfection, cells were collected by trypsinization and moved to a spinner flask that contain 200 ml of serum free media BalanCD HEK293 (IrvineScientific), then cultured at 37 °C, 5%CO_2_ under constant agitation with magnetic stirrer for three days. Culture supernatants were directly purified with 500 μl of protein G Sepharose (GE Healthcare). After elution from protein G Sepharose twice with 50 μl of 100 mM Glycine pH 2.7, protein solutions were neutralized with 10 μl of 1 M Tris-HCl (pH 8.5), then dialyzed twice against 100 ml of PBS.

### Immunoprecipitation and Immunoblotting

Recombinant proteins were mixed with the indicated combinations at the indicated amounts, and incubated for 10 to 12 hours at 4 °C under rotation. The protein mixtures were further incubated after addition of 1 μg of the appropriate antibodies for 4 hr at 4 °C, then incubated after addition of 5 μl of protein G-Sepharose for 1 hr at 4 °C. Immunoprecipitates were washed five times with wash buffer (140 mM NaCl, 0.1% Triton X-100, 1 mM EDTA, 10 mM Tris (pH 8.0)). Proteins were dissoleved in SDS-PAGE sample buffer, then separated by SDS-PAGE and electrotransferred to an Immobilon-P PDVF membrane (MERCK-Millipore). Membranes were hybridized with the indicated antibodies, then visualized with an ImmunoStar-LD ECL substrate (WAKO).

### Protein G Sepharose pull-down assay

Recombinant proteins were mixed with the indicated combinations at the indicated amounts, and incubated for 10 to 12 hours at 4 °C under rotation. The protein mixtures were further incubated after addition of 5 μl of protein G Sepharose for 2 hr at 4 °C under rotation. For the analysis of CTLA-4 binding to CD80, HA-RGMB-ECD-Fc and/or V5-CD80-ECD-Fc proteins were first incubated with 1 μl (binding capacity for 2 μg of IgG) of protein G Sepharose for 10–12 hours at 4 °C under rotation. Then the RGMB and/or CD80-loaded protein G Sepharose were further incubated after addition of 1 μg of Flag-memCTLA4-ECD or Flag-sCTLA-4 for 4 hours at 4 °C under rotation. Pulled-down samples were washed five times with wash buffer (140 mM NaCl, 0.1% Triton X-100, 1 mM EDTA, 10 mM Tris (pH 8.0)). Proteins were dissoleved in SDS-PAGE sample buffer, then separated by SDS-PAGE and electrotransferred to an Immobilon-P PDVF membrane (MERCK-Millipore). Membranes were hybridized with the indicated antibodies, then visualized with an ImmunoStar-LD ECL substrate (WAKO).

### Analysis of transcriptome datasets

The SRA files were downloaded from the SRA database. Then, the SRA files were converted to FASTQ format. FASTQ files were “purified” and trimmed with fastq_quality_trimmer commands, then mapped to the corresponding genomes with Tophat2. FPKM were determined with Cufflinks. For the analysis of GSE datasets, corresponding series_matrix.txt files were downloaded, and the expression levels of the intended genes were analyzed. Expression levels of mRNA encoding CTLA-4 variants in Treg cells and mDC cells were derived from RNA-seq data deposited in SRP006674 and SRP059735, respectively.

### CRISPRi knockdown of RGMB in Raji B cells

SgRNA expression cassettes were constructed by inserting a 20 nt sequence 5′-CTCGTCTCAGCAGTCGCTCA-3′, that target the proximal promoter of human *RGMB*, between the U6 promoter and the gRNA scafold sequence. The sgRNA expression cassettes, that was followed by EF-1α core promoter-driven mouse Thy1.1 reporter, were sub-cloned into a retroviral expression plasmid, thus constructing a “pRetroX-U6-promoter-RGMB-gRNA-EF-a-promoter-mThy1.1” plasmid. RGMB promoter sgRNA expressing retrovirus were produced using this plasmid. Retrovirus for expression of dCas9-KRAB-P2A-AcGFP fusion protein was independently produced, and co-transduced with the *RGMB* sgRNA expression retrovirus into Raji B cells. Raji B cells that co-express *RGMB* sgRNA and dCas9-KRAB were sorted according to Thy1.1 and AcGFP reporter gene expressions.

### Retroviral overexpression of RGMB in Raji B cells

The ORF that encodes full-length human RGMB, that was followed by internal ribosomal entry site (IRES) and tandem RFP (tdRFP), was sub-cloned into a retroviral expression plasmid. Retrovirus for RGMB expression was produced using this plasmid. Raji cells transduced with the RGMB expression retrovirus were sorted according to tdRFP expression.

### CTLA-4 blockade bioassay with Jurkat T and Raji B cells

First, Raji B cells (1 × 10^5^ cells) were incubated with 3 or 10 μg/ml of CTLA-4-Fc or sCTLA-4 recombinant proteins for 30 min in 100 μl of RPMI1640 plus 10% FBS media in round-bottomed 96-well plates. Then, Jurkat cells (5 × 10^4^ cells) and 100 ng/ml of anti-CD3ε antibodies (OKT3) were added in this sequence. Twenty four hour after, culture supernatants were collected and IL-2 concentration was analyzed by ELISA with Human IL-2 Ready-SET-Go! kit (eBioscience), according to the manufacture’s protocol.

### Analysis of TCGA datasets

TCGA RNA-seq data of thirty-three non-hematological cancer were downloaded from the TCGA website (http://gdac.broadinstitute.org/). Spearman’s correlation coefficients and *p*-values were determine with cor.test function of R. In the analysis of the correlation between RGMB mRNA expression levels and T cell populations (CD4 naive, CD8, CD4 resting memory, and CD4 activated memory) in different non-hematological tumor types, the percentages of T cell populations, among twenty-two leukocyte compositions, were estimated by CIBERSORT of the TCGA RNA-seq dataset. We used 1,000 permutation and disabled quantile normalization.,

### Estimation of expression levels of sCTLA-4

*CTLA4* exon2:exon3 (chr2:204735656:+,chr2:204736101:+) and exon2:exon4 (chr2:204735656:+,chr2:204737431:+) junction reads were obtained from rnaseqv2__illuminahiseq_rnaseqv2__unc_edu__Level_3__junction_quantification__data.data.txt files in TCGA database. Samples which have more than 1 read at both exon2:exon3 and exon2:exon4 junctions were pooled from 33 non-hematologic tumor samples and used in further analysis (n = 521). Read counts for CTLA4 exon2 (chr2:204735309-204735656:+) and exon3 (chr2:204736101-204736210:+) bodies were obtained from rnaseqv2__illuminahiseq_rnaseqv2__unc_edu__Level_3__exon_quantification__data.data.txt files. Correlation determination was performed with cor.test function of R. Then, sCTLA-4 expression levels were determined for each tumor subtype as: expression levels of total *CTLA4* (obtained from rnaseqv2__illuminahiseq_rnaseqv2__unc_edu__Level_3__RSEM_genes_normalized__data.data.txt files) multiplied by (total exon2 reads - tolal exon3 reads)/total exon2 reads.

### Construction of Rgmb-expressing bone marrow derived dendritic cells

Bone marrow cells were collected from the tibia and femur of wildtype C57BL/6 J mice. Cells were cultured for 3 d at a density of 2 × 10^5^ cells/ml with mouse GM-CSF (PeproTech, 20 ng/ml), in DMEM containing 10% FBS. At 3 d of culture, cells were infected by centrifugation at 2,500 rpm for 2 h at 35 °C with a control retrovirus expressing GFP or with Rgmb- or Rgmb-ΔCtla-4-expressing retrovirus, whose expression can be monitored by IRES-driven expression of GFP, in a solution containing polybrene (2 μg/ml). After infection, the retroviral supernatant was removed and replaced with fresh growth media (20 ng/ml GM-CSF in DMEM containing 10% FBS). At 5 d after infection, non-adherent and loosely adherent cells in the culture supernatant were harvested and stained with PE-Cy7 conjugated anti-mouse CD11c antibodies, then CD11c^+^GFP^+^ cells were sorted as transgene-positive bone marrow derived dendritic cells. Cells were further incubated in 0.1 μg/ml of LPS in DMEM containing 10% FBS for 24 h and supplied for mixed lymphoid reaction with CD8^+^ T cells.

### Mixed lymphoid reaction

First, BMDCs generated as described above (5 × 10^3^ cells) were incubated with 3 or 10 μg/ml of mouse Ctla-4-Ig or sCtla-4 recombinant proteins for 30 mij1n in 100 μl of RPMI1640 containing 10% FBS in round-bottomed 96-well plates. Then, naive CD8^+^ T cells (CD8^+^CD62L^+^, 5 × 10^4^ cells) and 1 μg/ml of anti-CD3ε antibodies (clone 145-2C11) were added in this sequence. 6 d after, total cells were analyzed for *Gzmb* and *Prf1* mRNA expression and Granzyme B protein expression, with RT-qPCR and flow cytometry analysis, respectively.

### Statistical analysis

*p* values were calculated with Graphpad Prism software and R. *p* values of less than 0.05 were considered significant. All error bars in graphs represent SEM calculated at least three replicates.

## Supplementary information

Supplementary Information

## Data Availability

The datasets generated during and analyzed during the current study are available from the corresponding author on reasonable request.
