# Supplementary material for: RGMB enhances the suppressive activity of the monomeric secreted form of CTLA-4
Source: Sci Rep. 2019 May 6;9:6984. doi: 10.1038/s41598-019-43068-y (PMC6502797; doi:10.1038/s41598-019-43068-y)
Supplement: Supplementary file 1 — Supplementary Information [file 41598_2019_43068_MOESM1_ESM.pdf]

Supplementary Information:

RGMB enhances the suppressive activity of the monomeric secreted form of CTLA-4

Takashi Sekiya* and Satoshi Takaki

*Correspondence: Corresponding Author: lb-sekiya@hospk.ncgm.go.jp

Unprocessed immunoblot images

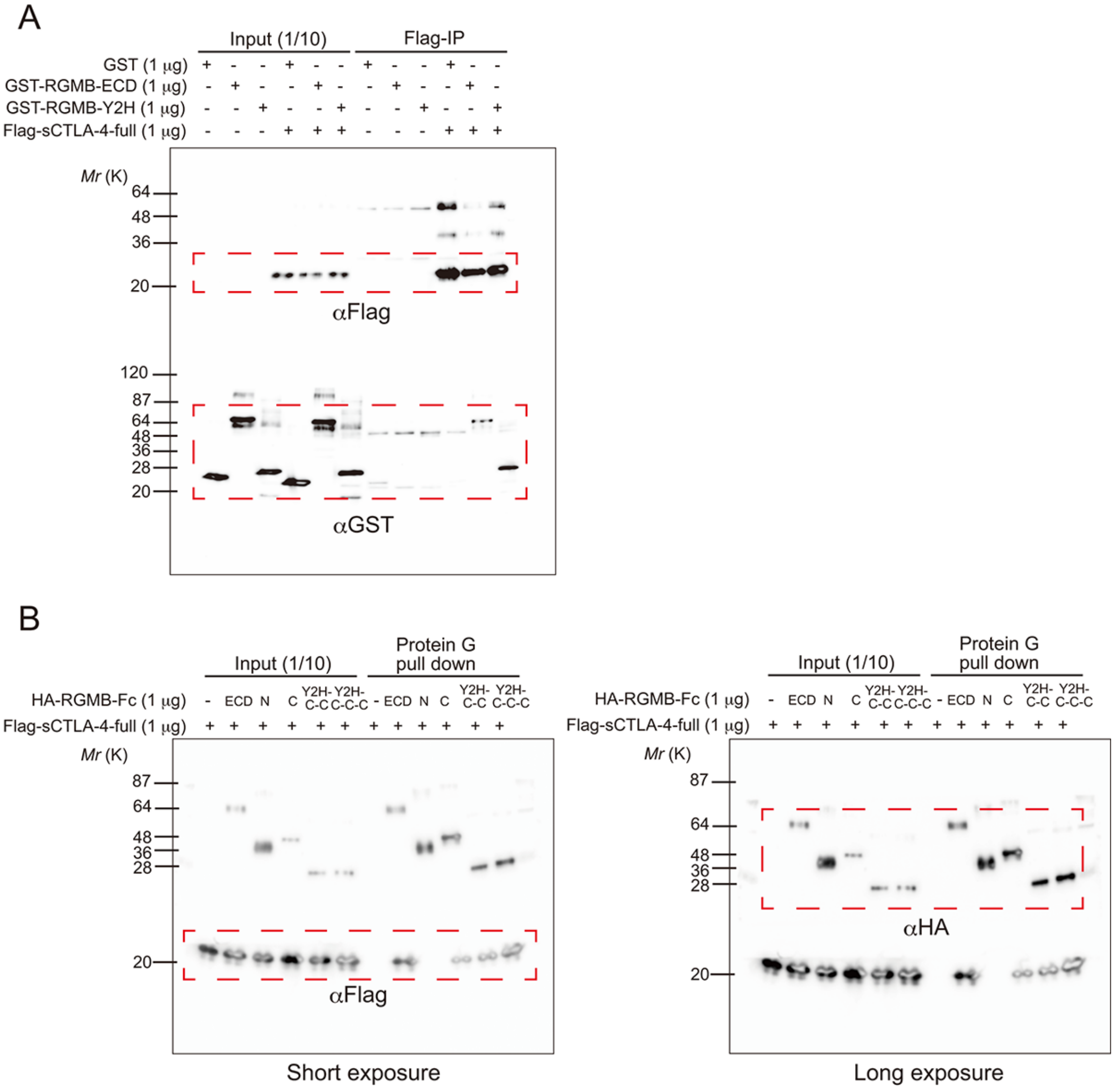

Supplementary Figure S1: Uncropped versions of all Western blots shown in Fig. 1. (A),(B) shows uncropped versions of Western blots shown in **Fig. 1A** and **1C**, respectively. Cropped areas for the main figures are indicated with the dashed lines.

Unprocessed immunoblot images

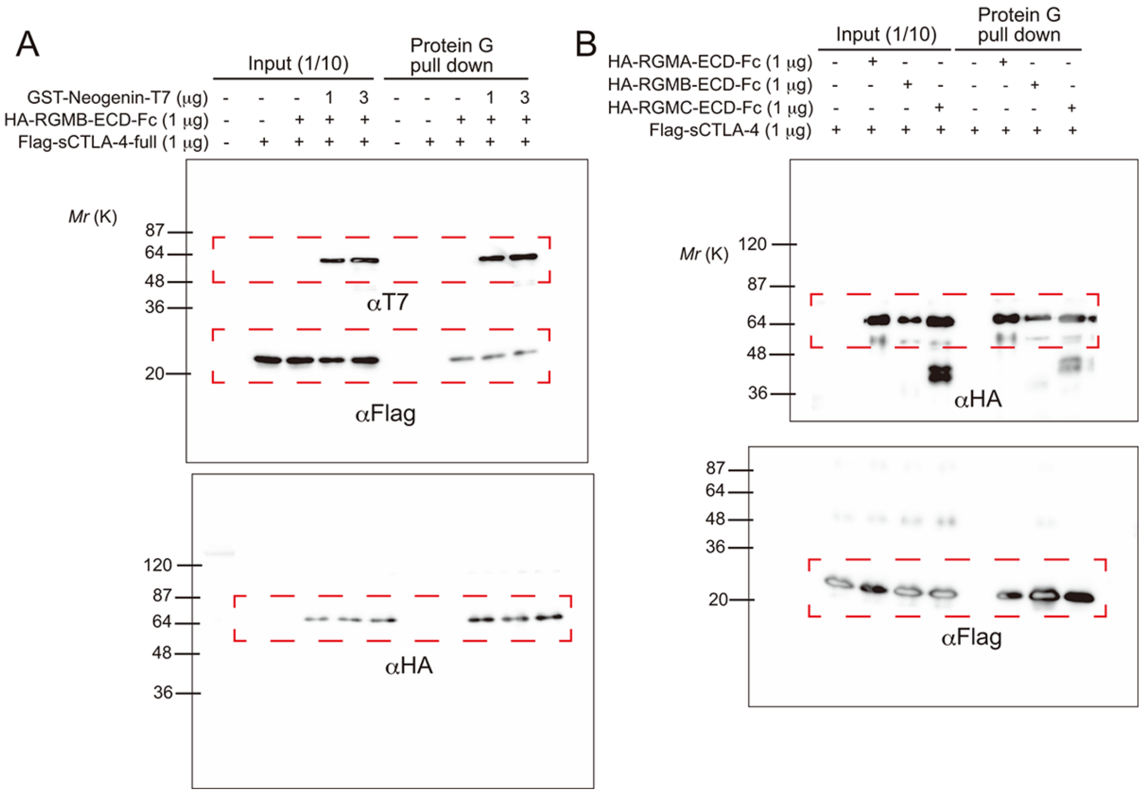

Supplementary Figure S2: Uncropped versions of all Western blots shown in Fig. 2.
(A),(B) shows uncropped versions of Western blots shown in **Fig. 2A** and **2C**, respectively. Cropped areas for the main figures are indicated with the dashed lines.

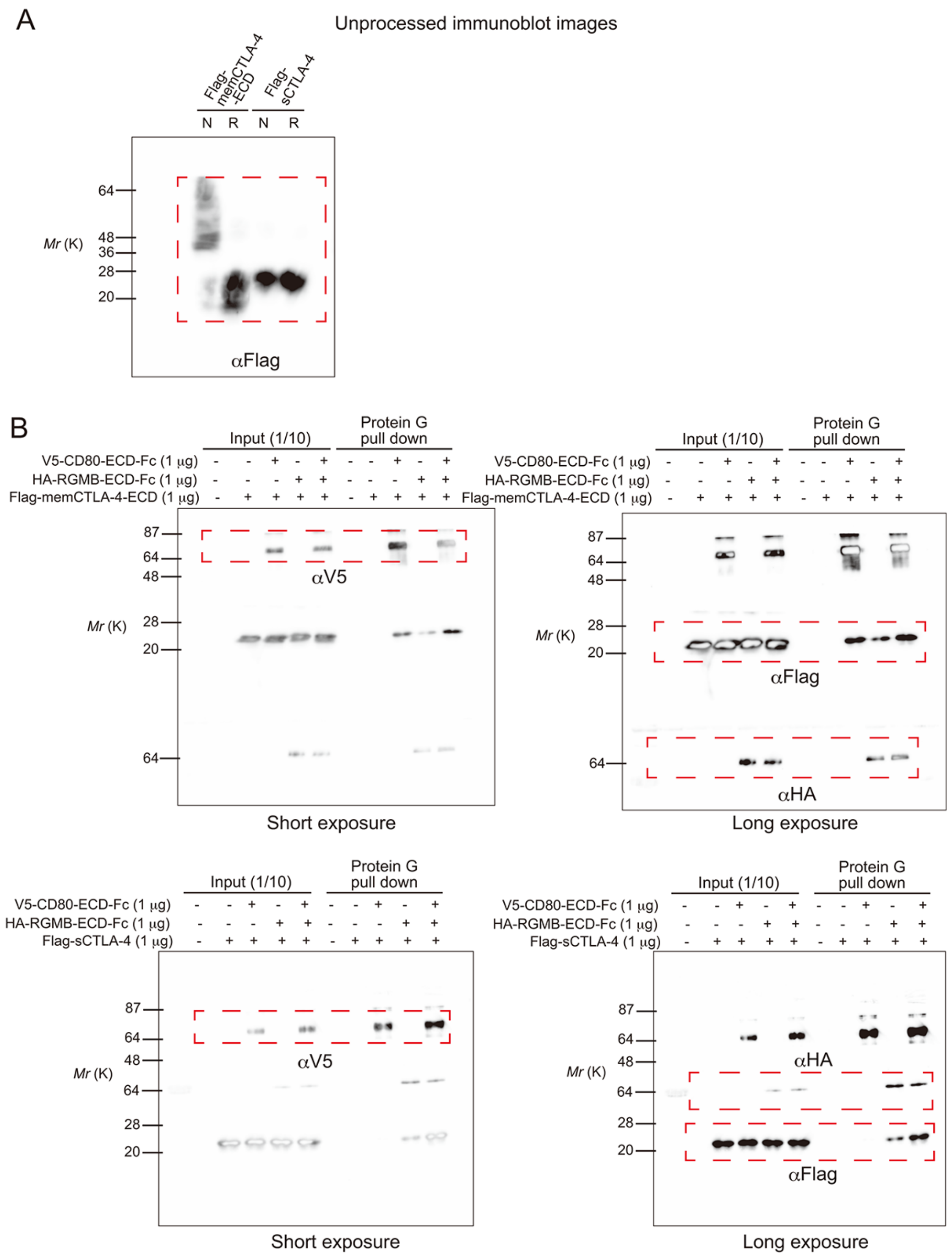

Supplementary Figure S3: Uncropped versions of all Western blots shown in Fig. 4. **(A),(B)** shows uncropped versions of Western blots shown in **Fig. 4A** and **4B**, respectively. Cropped areas for the main figures are indicated with the dashed lines.

Unprocessed immunoblot images

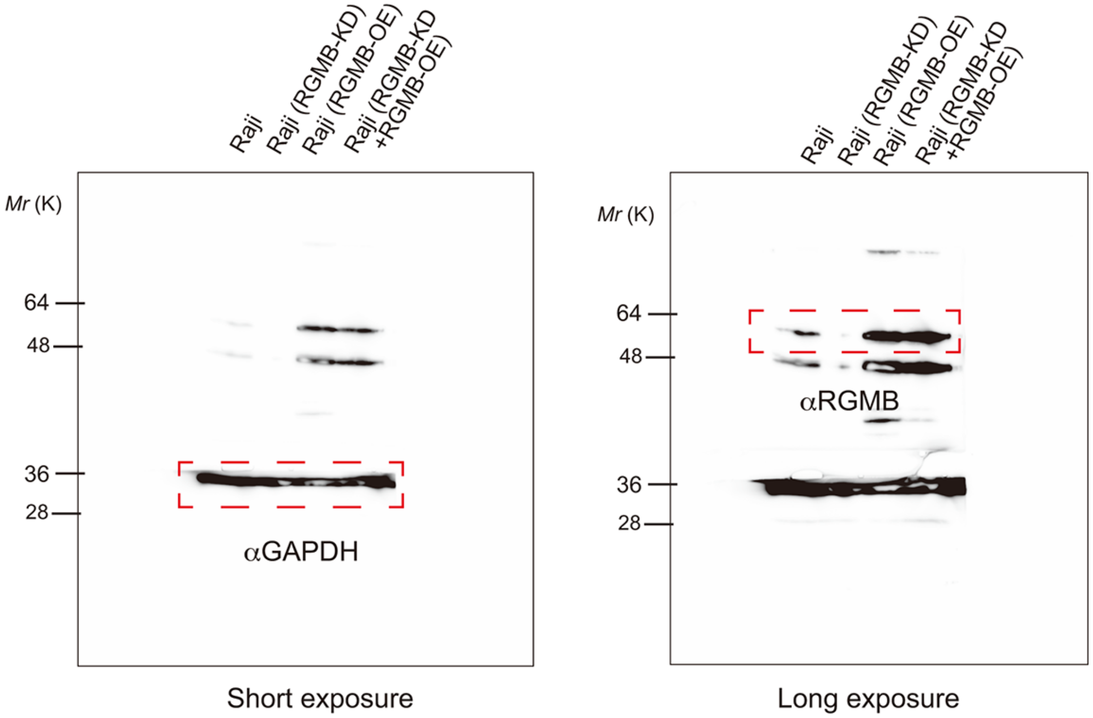

Supplementary Figure S4: Uncropped versions of all Western blots shown in Fig. 5.

Unprocessed immunoblot images

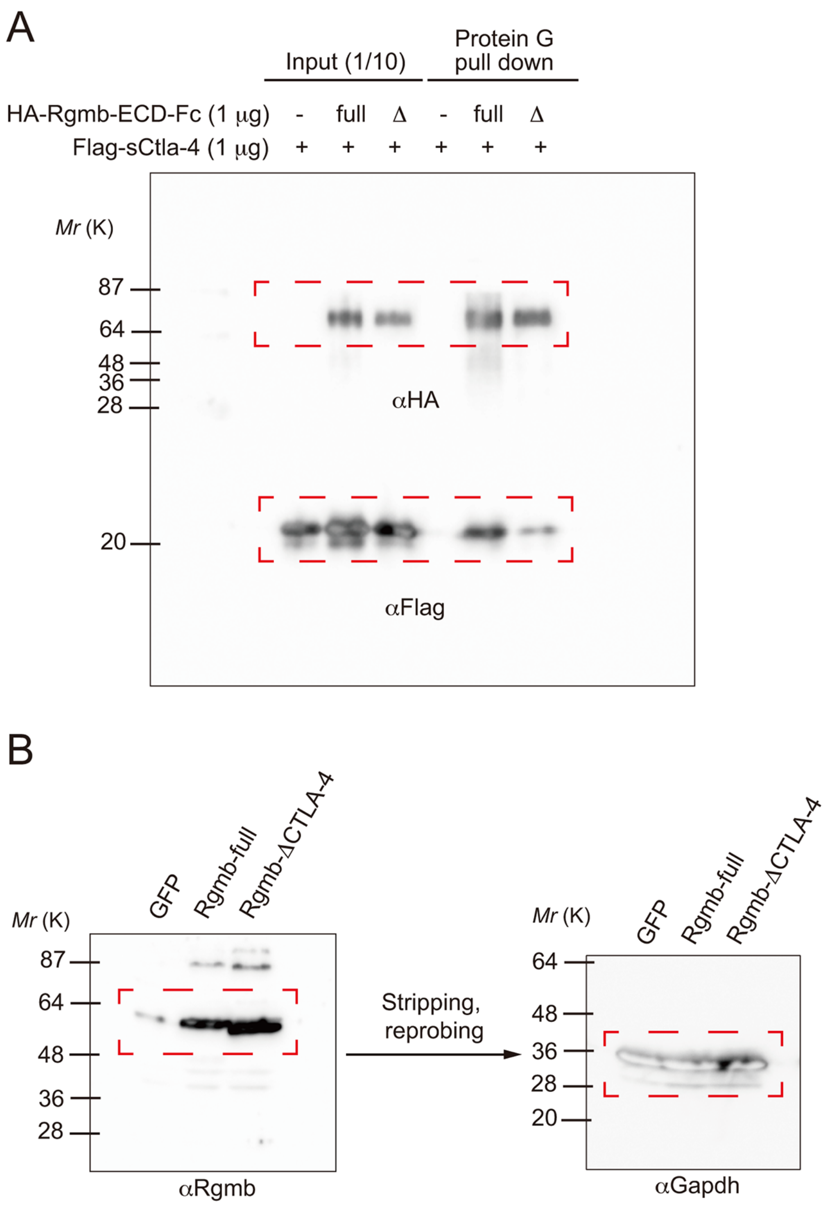

Supplementary Figure S5: Uncropped versions of all Western blots shown in Supplementary Fig. S7.

(A),(B) shows uncropped versions of Western blots shown in **Supplementary Fig. S7B** and **S7E**, respectively. Cropped areas for the main figures are indicated with the dashed lines.

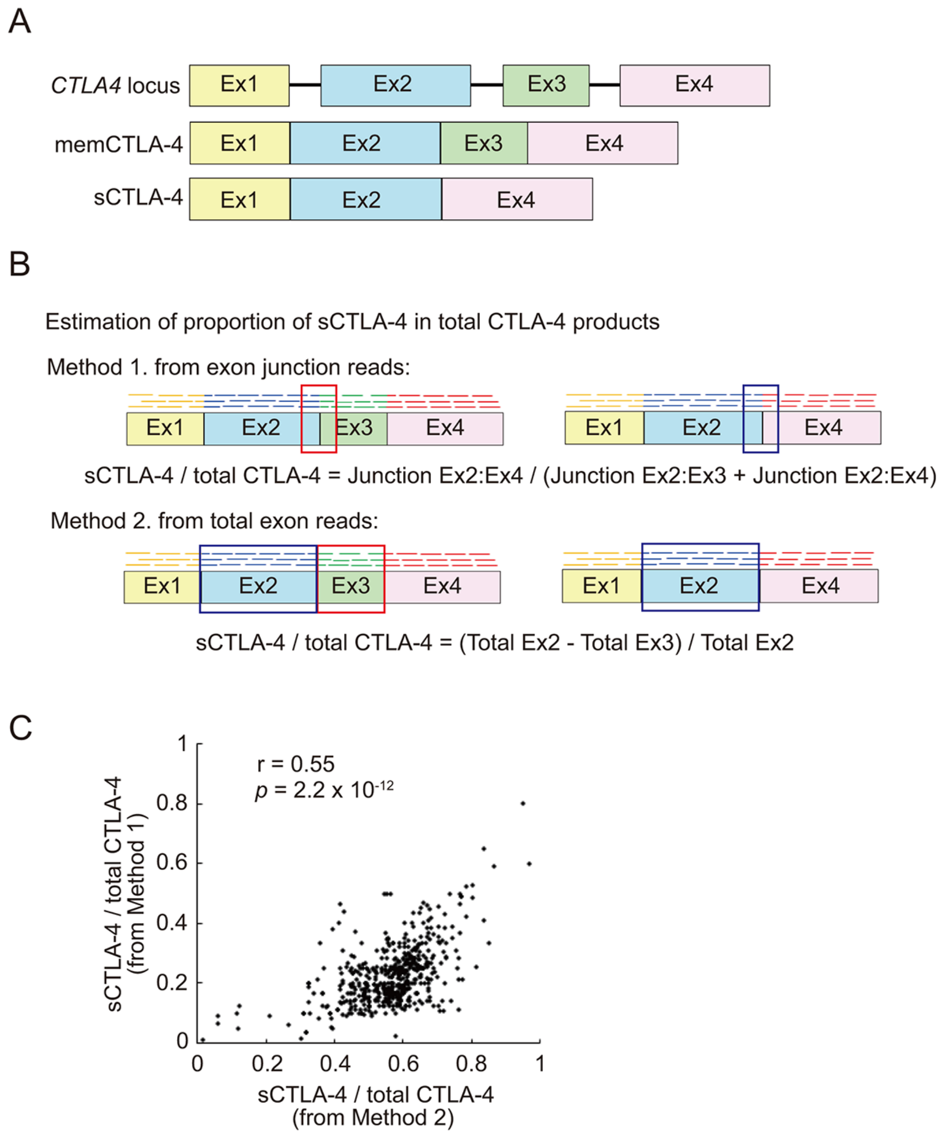

Supplementary Figure S6: A method to determine the expression levels of sCTLA-4 in TCGA datasets.

(A) Exon-intron structure of *CTLA4*. **(B)** Schematics of the methods that determine the proportion of sCTLA-4 in total CTLA-4 products. **(C)** Correlation between the proportions of sCTLA-4 in total CTLA-4 products determined by the Method 1 and the Method 2 shown in **(B)**. Samples which have more than 1 read at both exon2:exon3 and exon2:exon4 junctions were pooled from 33 non-hematologic tumor samples and used in this analysis (n=521). Spearman's correlation coefficient and *p* value are shown.

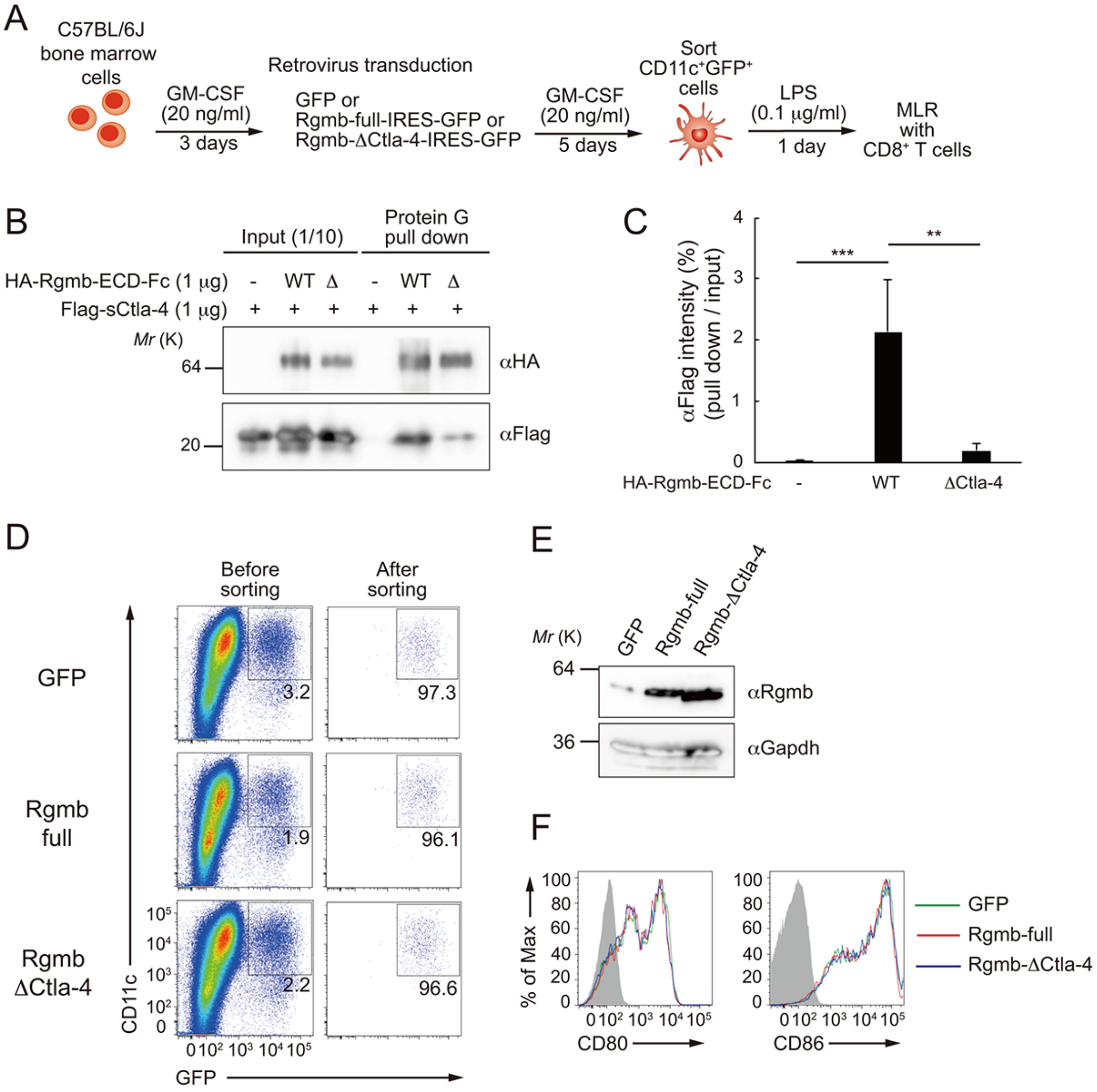

Supplementary Figure S7: Construction of Rgmb-overexpressing BMDCs.

(A) Schematic of experiments shown in **Fig. 6**. **(B)** Association of mouse Rgmb with mouse sCtla-4 *in vitro*. Mammalian cell-produced full-length sCtla-4 (1 μg) was incubated with 1 μg of mammalian cell-produced extracellular domain of wildtype Rgmb (WT) or of deleted form of Rgmb which lacks a region corresponding to the CTLA-4 interaction domain of human RGMB (Δ). HA-Rgmb-ECD-Fc was pulled-down with protein G Sepharose, and the bound proteins were subjected to immunoblotting analysis with the antibodies indicated. Full-length blots are shown in **Supplementary Fig. S5A**. **(C)**

Quantification of the results in **(B)**. Intensities of the bands of pulled-downed Flag-sCtla-4, relative to the intensities of the input bands, are shown. **(D)** Flow cytometry of CD11c and GFP expression in BMDCs, which were transduced with retrovirus which express GFP, full length Rgmb, or deleted Rgmb which lacks the Ctla-4 interaction region, whose expression can be monitored by IRES-mediated co-expression of GFP. Indicated areas in the left panels were sorted and supplied for MLR experiment shown in **Fig. 6**. **(E)** Western blot analysis of the expression levels of Rgmb and Gapdh in the indicated cells. Full-length blots are shown in **Supplementary Fig. S5B**. **(F)** Flow cytometry of the indicated molecules in the indicated cells. Shaded area represent the isotype-matched control. Scores show means $\pm$ SD of data obtained from three independent biological replicates. *$p<0.05$ (one-way ANOVA with Bonferroni test).
